# Comment on: Effect of combined training on body image, body composition and functional capacity in patients with breast cancer: controlled clinical trial

**DOI:** 10.61622/rbgo/2024rbgo96

**Published:** 2024-12-04

**Authors:** Piyush Bharadwaj, Divya Khati, Simran Sunil Khutarkar, Sweta Verma

**Affiliations:** 1 Lovely Professional University Department of Physiotherapy Jalandhar Punjab India Department of Physiotherapy, Lovely Professional University, Jalandhar, Punjab, India.

Dear Editor,

I am writing to express my appreciation for the recent article titled "Effect of Combined Training on Body Image, Body Composition, and Functional Capacity in Patients with Breast Cancer: Controlled Clinical Trial," published online on June 20, 2023. The study provides crucial insights into the benefits of combined training for breast cancer patients, highlighting significant improvements in body image and functional capacity.^(1)^ The Comprehensive approach of integrating aerobic, resistance, and flexibility training over 12 weeks is particularly noteworthy. This multidimensional exercise regimen and its positive outcomes on patient health are valuable contributions to oncology rehabilitation.

However, I would like to offer several points to enhance the clarity and comprehensiveness of the study. In the introduction, while the authors mention that breast cancer and its treatment can generate significant changes in body appearance and functionality, including alopecia, surgical scars, breast removal, and reduced strength and cardiopulmonary capacity, they do not specify and clarify which stage patients were included in the study and how the different stages of breast cancer might affect the outcomes of the exercise interventions.^(2)^ Including a discussion on how the severity of the disease could influence the effects of combined training on body image, body composition, and functional capacity would provide more nuanced insights and help tailor interventions more effectively for patients at various stages of breast cancer.

In the first paragraph of the methods section, one notable omission is the lack of detail regarding pre-assessment procedures. Pre-assessment is crucial for establishing baseline measurements and ensuring the accuracy and reliability of post-intervention comparisons. Including a thorough description of the pre-assessment process would strengthen the study's methodology and provide a clearer understanding of the intervention's impact. This would also help identify any pre-existing conditions that might affect the outcomes of the exercise regimen. Additionally, the methods section does not specify which guidelines were followed for the exercise prescription such as those from the American College of Sports Medicine (ACSM). Specifying the guidelines used would add credibility to the study and provide a clear framework for healthcare professionals looking to implement similar programs.^(3)^

Furthermore, the study's methodology could benefit from a more detailed description of the exercise protocols used. This information is essential for replicating the study and for healthcare professionals wishing to implement similar programs. In the results section, the study evaluates the impact of a 12-week combined aerobic and resistance training regimen on 26 breast cancer patients, with significant improvements noted in body image (reduction in the limitation dimension, p=0.036) and functional capacity (increased VO2max, p<0.001, and strength in both arms, right arm, p=0.005; left arm, p=0.033). The literature indicates that BMI, waist-hip ratio, waist circumference (WC), and fat-free mass are predictors of negative changes in body image, such as body dissatisfaction. While the results regarding functional capacity improvements are promising, discussing potential reasons behind the increase in waist circumference observed in both groups would be beneficial.^(4)^

This could include considerations of dietary intake, changes in visceral fat, or other physiological factors that might influence waist measurements despite overall improvements in physical fitness and body image. Understanding these factors is crucial to providing a comprehensive interpretation of the findings.

In conclusion, by addressing these aspects and refining methodology, future research can enhance the validity and generalizability of findings, contributing significantly to the field of oncology rehabilitation and ultimately improving the holistic care of breast cancer patients. I commend the authors for their valuable research and encourage further studies to build on these findings, exploring broader and more integrated rehabilitation strategies for cancer patients.
